# Supplementary material for: Do Egg Hormones Have Fitness Consequences in Wild Birds? A Systematic Review and Meta‐Analysis
Source: Ecol Lett. 2025 Mar 18;28(3):e70100. doi: 10.1111/ele.70100 (PMC11920385; doi:10.1111/ele.70100)
Supplement: Supplementary file 1 — FIGURES S1‐S5. [file ELE-28-0-s002.docx]

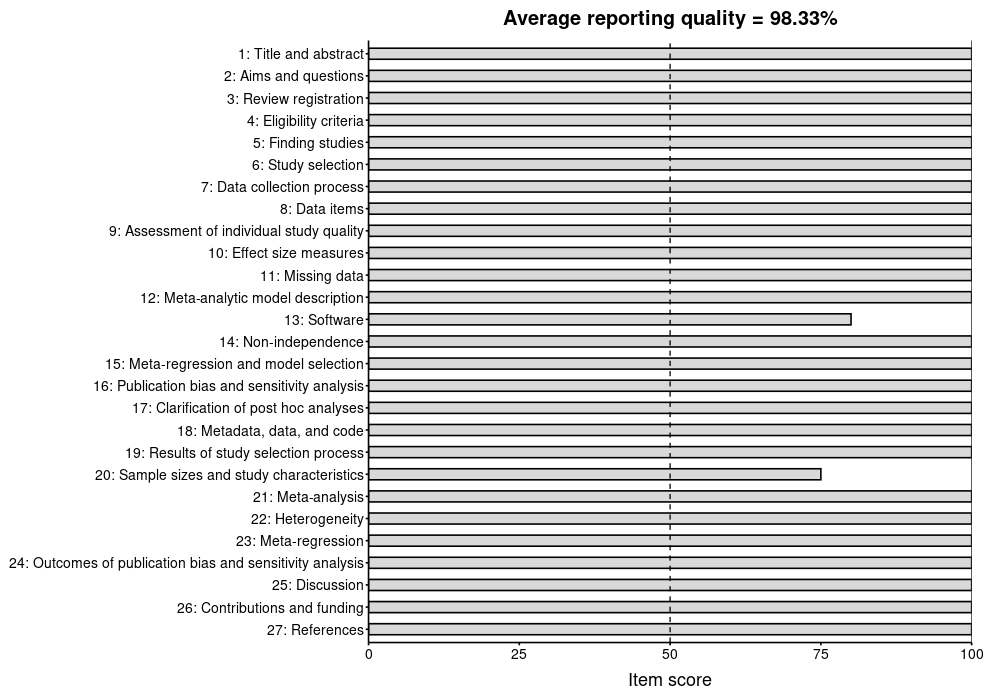
**Supplementary Figure 1.** Quality assessment of our systematic review and meta-analysis testing the extent to which prenatal maternal hormone deposition into eggs relates to fitness in wild birds. For it, we filled out the ‘Interactive PRISMA-EcoEvo Checklist’ (https://prisma-ecoevo.shinyapps.io/checklist; O’Dea *et al.* 2021).


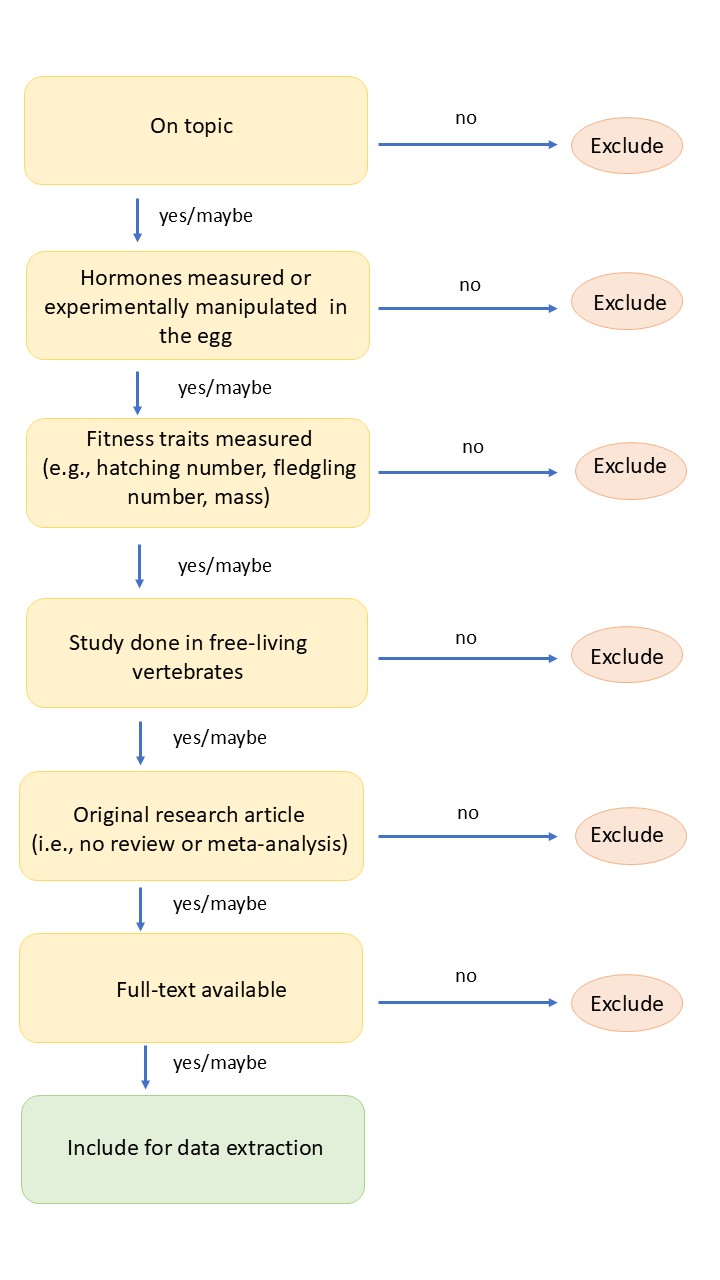
**Supplementary Figure 2.** Complete decision tree used for both title-and-abstract and full-text screening. Note that in our pre-registration (Mentesana *et al.* 2021), we had separate trees for title-and-abstract and full-text screening, but here we present a unified and complete version for clarity.

**Supplementary Figure 3.** PRISMA flow diagram summarizing our literature search. Of 80 eligible articles, we could not include 20 studies in the meta-analysis because articles reported incomplete information (e.g., missing sample sizes) and the authors did not provide such information despite our author contacting efforts (see “Materials and Methods” section), 1 article did not explore the
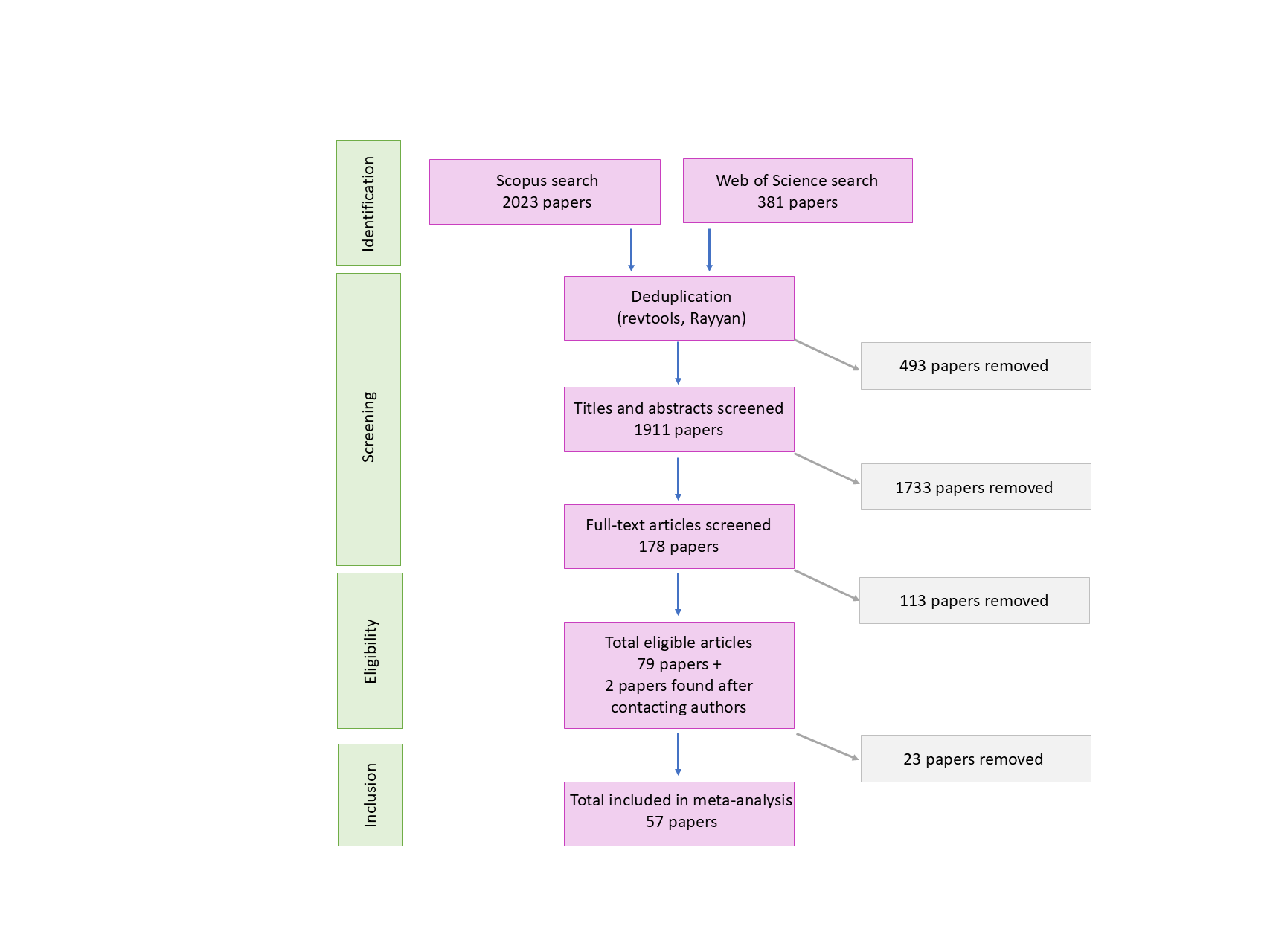
effect of a hormone *per se* but that of a hormone blocker, and 2 studies were conducted in turtles.

**Supplementary Figure 4.** Heterogeneity metrics and stratification for a meta-analysis testing the association between maternal egg hormones and fitness across 19 bird species. The heterogeneity is quantified using A) raw variance (𝜎^2^), B) source measure 𝐼^2^, C) magnitude measure 𝐶𝑉, and D) magnitude measure 𝑀, and stratified at phylogenetic (Phylo), non-phylogenetic among-species (Spp), among-population (Pop), among-laboratory (Lab), among- (Among) and within-study (Within) levels.


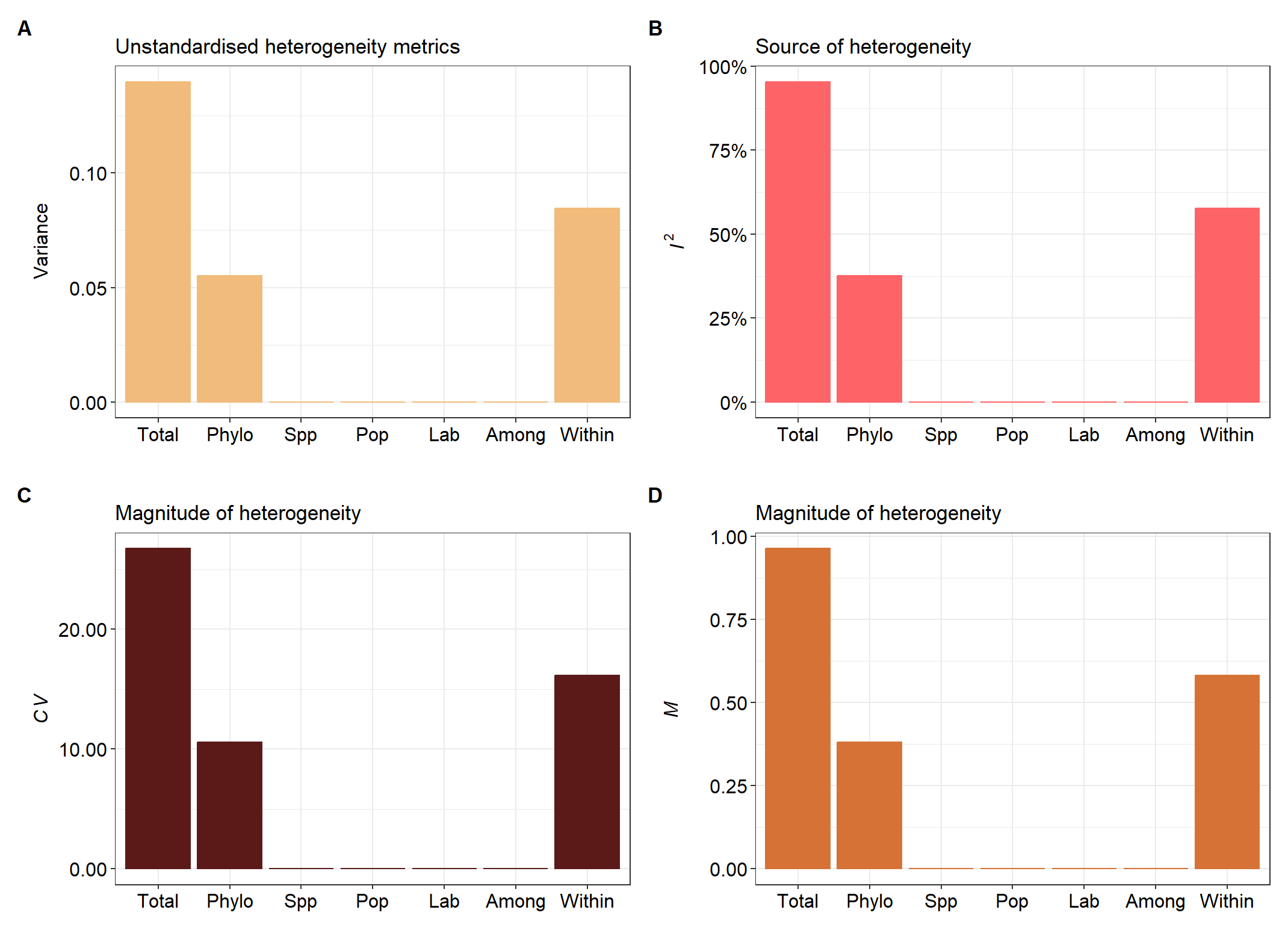


**Supplementary Figure 5.** Effect sizes obtained from the full dataset (considering both maternal and offspring fitness) using a phylogenetic multilevel meta-regression model with 'fitness proxies' as the sole moderator. Orchard plot showing mean estimates (circles with black outlines), 95% confidence intervals (thick whisker), 95% prediction intervals (thin whisker), and individual effect sizes scaled by their precision (coloured circles). k indicates the number of individual effect sizes and, in between brackets, the number of studies. Note that this model was not pre-registered and was conducted after obtaining the results to better understand them.


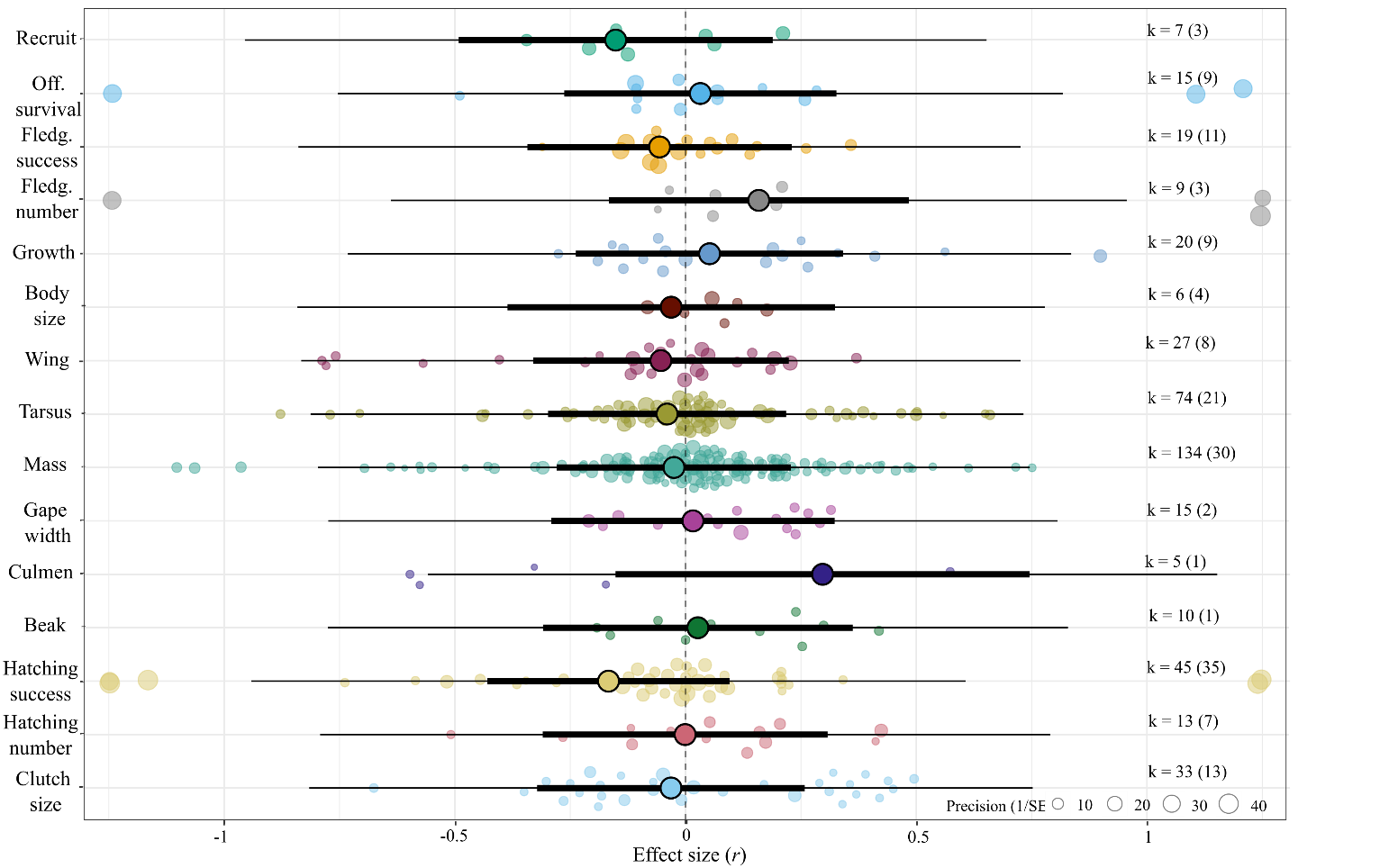


**References**

Mentesana, L., Hau, M., Adreani, M.N., D’amelio, P. & Sánchez-Tójar, A. (2021). Fitness consequences of egg hormones in the light of evidence synthesis. Retrieved from osf.io/st38j. *OSF*.

O’Dea, R.E., Lagisz, M., Jennions, M.D., Koricheva, J., Noble, D.W.A., Parker, T.H., *et al.* (2021). Preferred reporting items for systematic reviews and meta-analyses in ecology and evolutionary biology: a PRISMA extension. *Biol. Rev.*, 96, 1695–1722.
